# Removal of a Migrated Intrauterine Contraceptive Device Perforating the Terminal Ileum: A Case Report

**DOI:** 10.7759/cureus.29748

**Published:** 2022-09-29

**Authors:** Khalid Y Alharbi, Hossein A Filimban, Salman W Bafageeh, Aqeel S Binaqeel, Majed A Bayzid, Nabeel M Brasha

**Affiliations:** 1 Medicine, King Saud Bin Abdulaziz University for Health Sciences, College of Medicine, Jeddah, SAU; 2 Medicine, King Abdullah International Medical Research Center, Jeddah, SAU; 3 Obstetrics and Gynecology, Ministry of National Guard Health Affairs, College of Medicine, Jeddah, SAU; 4 Obstetrics and Gynecology, King Saud Bin Abdulaziz University for Health Sciences, College of Medicine, Jeddah, SAU; 5 Obstetrics and Gynecology, King Abdullah International Medical Research Center, Jeddah, SAU

**Keywords:** copper intrauterine device, terminal ileum, migration, perforation, intrauterine contraceptive device

## Abstract

We aim to present a rare case of a missing intrauterine contraceptive device (IUCD) that was found in the terminal ileum by laparoscopy and was managed initially by laparoscopy and then proceeded to laparotomy.

A 29-year-old female who had a copper IUCD inserted by a senior gynecologist presented to the clinic with pelvic pain and discomfort. She underwent laparoscopy for IUCD removal. Intraoperatively, the IUCD was discovered to be embedded in the terminal ileum, and therefore, laparoscopy was converted to an open laparotomy. The patient was readmitted multiple times because of abnormal fluid collection in the pelvic region, which was resolved finally by pigtail insertion.

This case sheds a light on the possibility of complications occurring in the medical field even if the practitioner is a senior gynecologist. Furthermore, missed IUCDs require thorough investigation and imaging to make an appropriate management plan to avoid serious complications.

## Introduction

Intrauterine contraceptive device (IUCD) insertion is one of the most effective methods of contraception, with a pregnancy prevention rate reaching 99% [1,2]. Uterine perforation is the most serious complication associated with IUCDs, but patients may be completely asymptomatic [2-4]. There are few articles describing IUCD transmigration and bowel perforation [4]. In this article, we present a rare case of a missing IUCD found in the terminal ileum by laparoscopy; initially, the case was managed by laparoscopy, but it was converted to laparotomy.

## Case presentation

A 29-year-old female, gravida 2, para 2+0, presented to the outpatient obstetrics clinic without an appointment complaining of pelvic pain and discomfort that had been occurring for one month. She had regular menses and denied any symptoms of abnormal uterine bleeding or vaginal discharge, changes in bowel habits, rectal bleeding, melena, dyspareunia, dysuria, or hematuria. A senior gynecologist inserted her copper IUCD one year ago; no difficulties occurred during the insertion. Her previous pregnancies were spontaneous vaginal deliveries. Otherwise, the patient had no significant medical, family, or social history.

On physical examination, the patient was conscious, alert, and oriented with stable vital signs. Her body mass index was 20.2, and her systemic review was normal. The IUCD thread was not visible during the vaginal examination.

**Figure 1:**
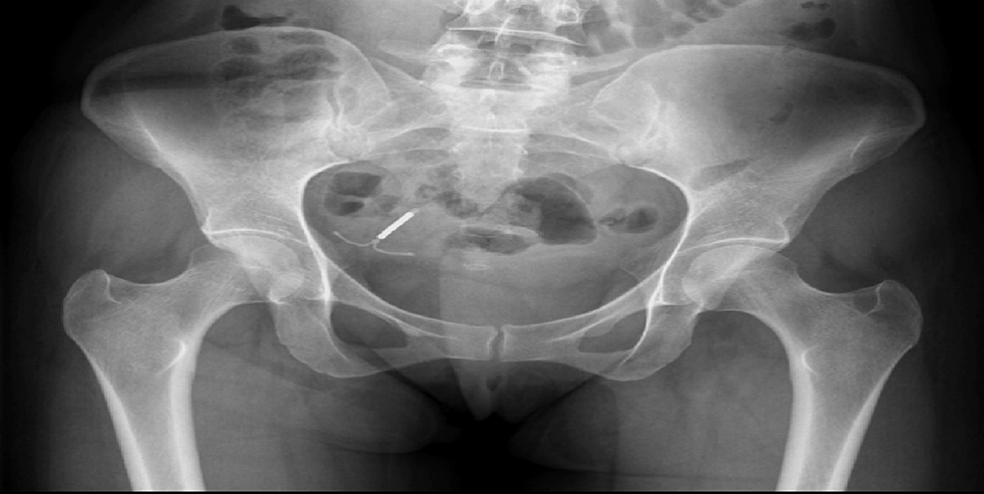
Intrauterine device in the right lower pelvic region

All laboratory test results were normal, and pregnancy was excluded. A pelvic transvaginal ultrasound revealed an IUCD that had migrated to the right adnexa adjacent to the right ovary. An X-ray showed the IUCD in the right lower pelvic region (Figure 1).

Subsequently, the patient was informed of the migrated IUCD and of the risk of a right salpingo-oophorectomy in case of bleeding; she was booked for an explorative laparoscopy and IUCD removal after consent was obtained.

In the operating room (OR), a panoramic view through the laparoscope indicated a normal uterus and left adnexa, while the right adnexa had moderate adhesions to the bowel and omentum. The IUCD was completely embedded in the terminal ileum. The general surgical team was involved, and they removed the IUCD from the bowel by making an initial incision using cautery followed by retraction of the IUCD. Due to the limited expertise of the on-call general surgeon, the operation was converted to an open laparotomy for ileocecal resection and anastomosis with no intraoperative spillage of bowel content. A new copper IUCD was inserted intrauterinely under direct visualization. The patient tolerated the procedure well and was moved to the recovery room in stable condition.

**Figure 2:**
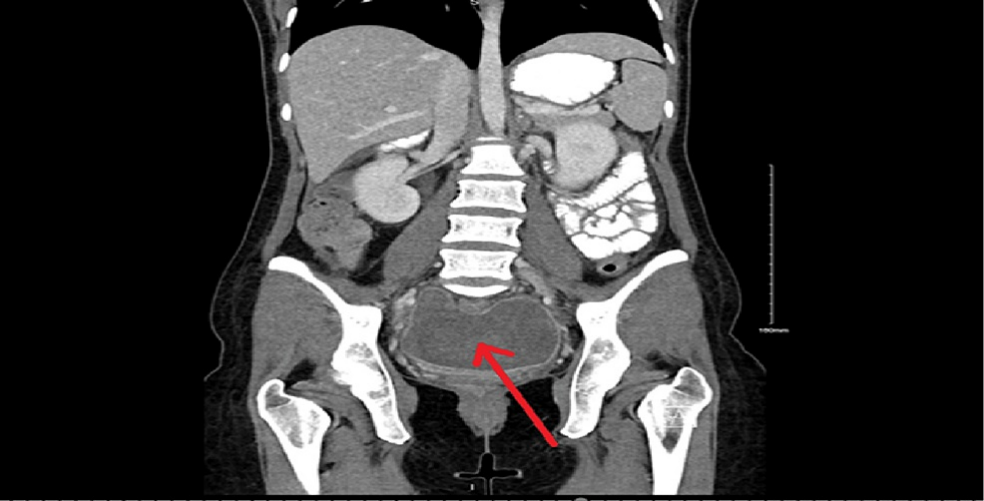
CT coronal view demonstrating a pelvic collection measuring 11.7×6.7×11.1 cm (red arrow) CT: computed tomography

The patient’s surgical and postoperative outcomes were normal, and she was discharged without complications on the second day post-operation. The removed IUCD demonstrated no bacterial growth. However, the resected bowel demonstrated ulceration with focal transmural inflammation (acute and chronic) and prominent Peyer’s patches (reactive lymphoid hyperplasia). The patient was readmitted to the emergency room (ER) for the first time five days postoperatively complaining of a fever (38.2°C) and right lower quadrant (RLQ) abdominal pain that had lasted for two days. The patient underwent multiaxial enhanced computed tomography (CT) to examine the anastomotic site. There was no evidence of oral contrast leakage from the anastomosis. However, a small area of loculated fluid measuring 1.4×0.8 cm was observed on the right side of the pelvis around the surgical sutures. The patient was started on empiric antibiotics for a total of 10 days (metronidazole 500 mg and cefuroxime 500 mg) on suspicion of early abscess formation, and the CT scan was repeated after five days. The CT results demonstrated the development of a large, slightly loculated peripherally enhancing pelvic fluid collection measuring 11.7×6.7×11.1 cm at intervals (Figure 2).

**Figure 3:**
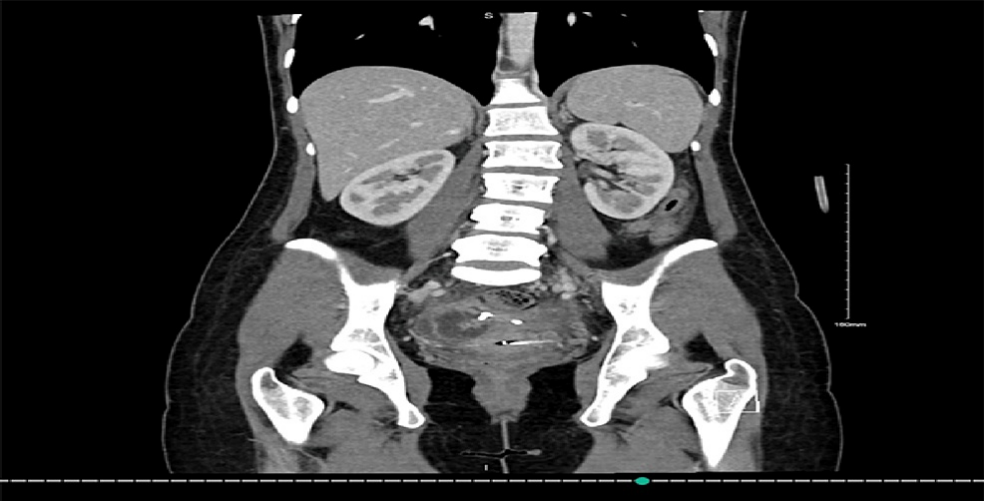
CT coronal view illustrating a marked decrease in the size of the previous collection CT: computed tomography

The patient was shifted to the OR as a level 2 emergency case for laparoscopic evacuation of the hematoma. She was discharged with antibiotics (metronidazole 500 mg and cefuroxime 500 mg) on the fourth day post-evacuation. Six days post-discharge, the patient was readmitted to the ER complaining of fever and RLQ colicky abdominal pain, which was aggravated by movement and not relieved by medication. Another CT scan was conducted that illustrated a slight decrease in the size of the previous fluid collection; it now measured 9.1×5.8×8.8 cm. Furthermore, the left ovary was embedded in the fluid collection. The interventional radiology team planned evacuation of the fluid collection via pigtail catheter insertion. Six days after pigtail catheter insertion, a CT scan demonstrated a marked decrease in the size of the collection (Figure 3).

The following day, the pigtail drain was removed, and the patient was discharged after completing her course of antibiotics (metronidazole 500 mg and cefepime 1,000 mg).

## Discussion

IUCD insertion procedures are the most common causes of uterine perforation [2]. Recent studies indicated that nearly half of the cases were not recognized until more than one year after insertion [2]. There are two basic forms of IUCDs: copper-containing IUCDs and levonorgestrel-containing IUCDs (Mirena) [5]. Copper-containing IUCDs work primarily as spermicidal agents; this mode of action is mediated by local inflammatory responses of the uterus to the IUCD as a foreign body [6]. Uterine perforation occurs with a frequency rate between 0.4 and 1.6 per 1,000 insertions [4]. The postulated mechanism of uterine perforation could either be due to primary penetration or subsequent inflammation [4,7]. Although copper-containing IUCDs work by inducing inflammation, a large cohort study showed that there were no significant differences in the uterine perforation rates of copper and levonorgestrel IUCDs [8]. The identified risk factors for uterine perforation were breastfeeding, postpartum IUCD insertion, uterine malformation, and IUCD insertion by less experienced clinicians; however, the precise cause of uterine perforation has yet to be established [4,8].

The symptoms of uterine perforation vary depending on the location of the IUCD [3]. IUCD perforation into bowel structures may cause a triad of abdominal pain, fever, and diarrhea [4]. However, 85% of the reported cases in the literature were asymptomatic [3].

The sign leading to the suspicion that an IUCD is missing is the absence of IUCD threads [4]. This warrants the need for further investigation to locate the IUCD, and initial localization by ultrasonography is preferred [4]. IUCDs that are localized extrauterinely need additional types of imaging for detection [4,9]. Moreover, since the IUCD is radiopaque, abdominopelvic radiography helps locate extrauterine IUCDs and diagnose IUCD expulsion [9]. The most accurate methods of IUCD localization are CT and magnetic resonance imaging (MRI) because they also evaluate the presence of intra-abdominal complications, such as visceral perforation, abscess formation, and bowel obstruction [9].

Minimally invasive methods are recommended for removing migrated IUCDs depending on their locations [3]. For intra-abdominal IUCDs, the preferred treatment is laparoscopic removal; however, in some cases, especially when an IUCD has perforated the bowel, an open laparotomy is indicated [8,10]. A review by Gill et al. [10] showed that in cases of small or large bowel perforations, 13/19 (68%) cases were managed by an immediate switch to laparotomy; adhesions were the main reasons for laparotomies. In 2014, Rahnemai-Azar et al. [11] reported successful retrieval of an IUCD from the small intestine by laparoscopy. They attributed their success to the surgeon’s ability and the wound protector retraction device, which provided good visualization.

In this case, pregnancy was excluded initially, and imaging led to a diagnosis of IUCD migration. However, CT and MRI were not performed due to a lack of concerning symptoms. The operation began laparoscopically, but the IUCD was not located. By following the thread, the IUCD was found embedded in the terminal ileum. Therefore, the operation was converted to an open laparotomy for bowel resection and anastomosis.

## Conclusions

Although only one year had elapsed since the insertion of the IUCD without complications by a senior gynecologist, it was found embedded in the terminal ileum, despite a pelvic transvaginal ultrasound indicating its location in the right adnexa. Furthermore, the on-call surgeon’s lack of experience resulted in the surgery being shifted from laparoscopy to laparotomy with bowel resection and anastomosis. Moreover, the patient was readmitted multiple times because of this event. This case sheds light on the possibility of complications whether a medical practitioner is a senior gynecologist or junior resident. It also emphasizes the importance of a thorough investigation of a missing IUCD to avoid more severe complications. Lastly, migration of IUCD and perforation of adjacent structures should always be mentioned during patient counseling.
